# Intelligence

**Published:** 1830-10

**Authors:** 


### INTELLIGENCE.

### APOTHECARIES' HALL, September 1830.

***Regulations to be observed by Students whose Attendance on Lectures shall commence on or after the 1st of January, 1831.****

Every candidate for a certificate to practise as an Apothecary, will be required to produce testimonials of having served an apprenticeship† of not less than five years to an apothecary:

Of having attained the full age‡ of twenty-one years:

Of good moral conduct:§ and

Of having devoted at least two years to an attendance on lectures and hospital practice.

*Course of Study.* The candidate must have attended the following courses of Lectures:||

Chemistry, two courses; each course consisting of not less than forty-five lectures.

Materia Medica and Therapeutics, two courses; each course consisting of not less than forty-five lectures.

Anatomy and Physiology, two courses; Anatomical Demonstrations, two courses; of the same extent as required by the Royal College of Surgeons of London.

Principles and Practice of Medicine, two courses; each course consisting of not less than forty-five lectures, to be attended subsequently to the termination of the first course of lectures on Chemistry, Materia Medica, and Anatomy and Physiology.

Botany, one course.

Midwifery, and the Diseases of Women and Children, two courses; Forensic Medicine, one course; to be attended during the second year.

Students are moreover recommended diligently to avail themselves of instruction in Morbid Anatomy.

The candidate must also have attended for twelve months, at least, the Physician's Practice at an Hospital containing not less than sixty beds, and where a course of Clinical Lectures is given; or for fifteen months at an Hospital wherein Clinical Lectures are not given; or for fifteen months at a Dispensary¶ connected with some Medical School recognized by the Court. The whole of such attendance to be subsequent to the first year of attendance on lectures.

* Students who are at present pursuing their medical studies, and those who may begin to attend lectures at the commencement of the next medical session, (viz. October,) will be received as candidates for examination by complying with the Regulations heretofore published.*

† The apprenticeship must have been served with a person legally qualified to practise as an apothecary, either by having been in practice prior to or on the 1st of August, 1815, or by having received a certificate of his qualification from the Court of Examiners.

‡ As evidence of age, a copy of the baptismal register will be required in every case where it can possibly be procured.

§ A testimonial of moral character from the gentleman to whom the candidate has been an apprentice, will always be more satisfactory than from any other person.

|| The lectures required in each course respectively, must be given on separate days.

¶ Certificates of attendance on the physician's practice at dispensaries will continue to be received until the 1st of January, 1833, from all such as have heretofore been admitted, but after that time the present regulation will be strictly adhered to.

* Vide London Med. and Phys. Journal for November 1829, p. 472.

The testimonials of attendance on Lectures, and Hospital Practice, must be given on a printed form, with which Students may be supplied, on application, at the under-mentioned places.

In London, at the beadle's office, at this Hall.

In Edinburgh, at Messrs. Mac Lachlan and Stewart's, booksellers.

In Dublin, at Messrs. Hodges and Smith's, booksellers.

In the provincial towns, where there are Medical Schools, at the Hospital, or from the teacher who keeps the register of the School.

Students are enjoined to observe that no other form of testimonial will be received; and that no attendance on lectures will qualify a candidate for examination, unless the teacher is recognized by the Court.

The teachers in Dublin, Edinburgh, Glasgow, and Aberdeen, recognized by the constituted medical authorities in those places respectively, are recognized by the Court.

*Registration.* A book* is kept at the Hall of the Society for the registration, at stated times, of the names of students, and the Lectures, Hospitals, or Depensaries they attend.

All students, in London, are required to appear personally, and to register the several classes for which they have taken tickets; and those only will be considered to have complied with the regulations of the Court whose names and classes in the register correspond with the testimonials of the teachers.

The book will be open for the registration during the first twenty-one days of the months of February, June, and October, from nine o'clock until two.

The Court also require students at the provincial Medical Schools to register their names in their own handwriting, and the classes they attend, with one of the teachers† in each respective school, within fourteen days from the commencement of each course of lectures, and those students only will be deemed to have complied with the regulations whose names are so registered.

Each student, at his first registration, will receive the printed form on which he is to obtain the certificates of his teachers.

The examination of the candidate will be as follows:

1. In translating parts of Celsus de Medicina, or Gregory Conspectus Medicinæ Theoreticæ, Pharmacopœia Londinensis, and Physicians' Prescriptions:
2. In Chemistry:
3. In Materia Medica and Therapeutics:
4. In Botany:
5. In Anatomy and Physiology:
6. In the Practice of Medicine.

*Notice.* Every person offering himself for examination must give notice in writing to the clerk of the Society on or before the Monday previously to the day of examination, and must also at the same time deposit all the required testimonials at the office of the beadle, where attendance is given every day, except Sunday, from nine until two o'clock.

Candidates will be admitted to examination in the order in which their names stand on the notice paper; and those neglecting to attend agreeably to their notice, will, upon a subsequent application, be placed at the bottom of the list.

* The book will be opened for the registration of those students whom these regulations affect, on the 1st of February, 1831.

† The students will be informed at each School, respectively, of the name of the teacher to whose care the register will be confided.

By the 22d section of the Act of Parliament no rejected candidate can be re-admitted to be examined until the expiration of six months from his former examination.

The Court meet in the Hall every Thursday, where candidates are required to attend at half-past four o'clock.

(By order of the Court,) JOHN WATSON, Secretary.

*Apothecaries' Hall; Sept.* 9, 1830.

The Act directs the following sums to be paid for certificates.

For London and within ten miles thereof, ten guineas.

For all other parts of England and Wales, six guineas.

Persons having paid the latter sum become entitled to practise in London, and within ten miles thereof, by paying four guineas in addition.

For an Assistant's certificate, two guineas.

For information relative to these Regulations, medical students are referred to Mr. Watson, who may be seen at his residence, 43, Berners street, between the hours of nine and ten o'clock every morning (Sunday excepted); and for information on all other subjects connected with the " Act for better regulating the Practice of Apothecaries," application is to be made to Mr. Edmund Bacot, clerk of the Society, who attends at the Hall every Tuesday and Thursday from one to three o'clock.

It is expressly ordered by the Court of Examiners, that no gratuity be received by any officer of the Court.

---

### THEATRE OF ANATOMY, GREAT WINDMILL STREET.

The lectures at this Theatre on Anatomy, Physiology, and Surgical Pathology, commence, as usual, on the 1st of October, and will be delivered solely by Mr. Mayo. Demonstrations will be given daily in the dissecting room.

### METEOROLOGICAL JOURNAL,

By Messrs. HARRIS and Co. Mathematical Instrument Makers, 50, High Holborn.

| | Moon. | Rain gauge. | Thermom. | | | Barometer. | | DeLuc's Hygrom. | | Winds. | | Atmospheric Variations. | | |
|---|---|---|---|---|---|---|---|---|---|---|---|---|---|---|
| | | | 9 a.m. | max. | min. | 9 a.m. | 10p.m. | 9 a.m. | 10p.m. | 9 a.m. | 10p.m. | 9 a.m. | 2 p.m. | 10p.m. |
| Aug. | | | | | | | | | | | | | | |
| 20 | | | 59 | 62 | 51 | 29.81 | 29.79 | 54 | 50 | NW | N | Fine | Fine | Fine |
| 21 | | | 56 | 63 | 50 | .84 | .82 | 48 | 49 | NW | NW | Cloudy | Fine | Fine |
| 22 | | | 55 | 66 | 60 | .82 | .86 | 50 | 55 | NW | WNW | Fine | Fine | Sho'ry |
| 23 | | .39 | 64 | 67 | 58 | .83 | .76 | 53 | 54 | WSW | SW | —— | —— | Fine |
| 24 | | | 65 | 66 | 55 | .72 | .71 | 54 | 56 | WSW | SW | —— | —— | Sho'ry |
| 25 | | | 57 | 69 | 57 | .64 | .57 | 56 | 55 | SW | SW | —— | —— | Cloudy |
| 26 | ☽ | | 59 | 65 | 52 | .67 | .71 | 53 | 52 | W | SW | Cloudy | —— | Cloudy |
| 27 | | | 56 | 62 | 54 | .63 | .32 | 52 | 53 | SW | SSE | Fine | —— | Rain |
| 28 | | | 57 | 60 | 52 | .25 | .44 | 55 | 54 | SW | SW | Rain | Sho'ry | Cloudy |
| 29 | | | 55 | 63 | 49 | .80 | .94 | 53 | 55 | W | W | Fine | Rain | Cloudy |
| 30 | | | 55 | 62 | 52 | 30.04 | 30.06 | 54 | 52 | WSW | W | Fine | Fine | Fine |
| 31 | | .78 | 59 | 66 | 53 | .11 | .12 | 52 | 50 | W | W | —— | —— | —— |
| Sept. | | | | | | | | | | | | | | |
| 1 | | | 57 | 67 | 53 | .14 | .12 | 49 | 50 | W | W | —— | —— | —— |
| 2 | ○ | | 59 | 67 | 55 | .03 | 29.85 | 50 | 52 | W | SW | —— | —— | Cloudy |
| 3 | | | 68 | 66 | 55 | 29.68 | .65 | 52 | 53 | W | W | —— | Cloudy | Fine |
| 4 | | | 60 | 64 | 55 | .78 | .79 | 53 | 53 | NW | NNW | —— | Fine | —— |
| 5 | | | 58 | 63 | 53 | .54 | .46 | 52 | 52 | WNW | SW | Rain | —— | Cloudy |
| 6 | | | 60 | 61 | 54 | .37 | .36 | 53 | 54 | SW | W | Cloudy | Sho'ry | Cloudy |
| 7 | | .40 | 60 | 62 | 54 | .55 | .70 | 55 | 57 | NW | NW | —— | Cloudy | Fine |
| 8 | | .03 | 58 | 61 | 51 | .84 | .85 | 57 | 56 | NNW | N | —— | Fine | —— |
| 9 | ☾ | | 58 | 59 | 53 | .72 | .60 | 55 | 58 | N | WSW | —— | Sho'ry | Rain |
| 10 | | | 56 | 60 | 47 | .50 | .57 | 60 | 57 | SW | WSW | Sho'ry | Fine | Fine |
| 11 | | | 55 | 59 | 51 | .62 | .58 | 56 | 53 | WSW | WSW | Fine | —— | —— |
| 12 | | .51 | 57 | 57 | 49 | .21 | .27 | 55 | 55 | S | SW | Sho'ry | Rain | —— |
| 13 | | | 57 | 62 | 52 | .34 | .26 | 55 | 54 | W | WSW | Fine | Fine | —— |
| 14 | | | 55 | 59 | 50 | .32 | .38 | 52 | 52 | W | SW | —— | —— | —— |
| 15 | | | 58 | 60 | 55 | .49 | .59 | 54 | 55 | SW | SSW | Cloudy | —— | Cloudy |
| 16 | | | 60 | 60 | 51 | .51 | .62 | 56 | 56 | WSW | SSW | Fine | —— | Fine |
| 17 | ● | | 55 | 57 | 53 | .51 | .48 | 56 | 57 | SE | ESE | Rain | —— | Foggy |
| 18 | | .54 | 55 | 59 | 48 | .44 | .67 | 60 | 60 | ESE | W | Fine | —— | Fine |
| 19 | | | 55 | 60 | 52 | .81 | .58 | 59 | 58 | SW | SW | —— | —— | Sho'ry |

The quantity of Rain fallen in August was 2 inches and 65.100ths.

